# Structural comparison of two ferritins from the marine invertebrate *Phascolosoma esculenta*

**DOI:** 10.1002/2211-5463.13080

**Published:** 2021-02-28

**Authors:** Tinghong Ming, Hengshang Huan, Chang Su, Chunheng Huo, Yan Wu, Qinqin Jiang, Xiaoting Qiu, Chenyang Lu, Jun Zhou, Ye Li, Xiurong Su

**Affiliations:** ^1^ State Key Laboratory for Managing Biotic and Chemical Threats to the Quality and Safety of Agro‐products Ningbo University China; ^2^ School of Marine Sciences Ningbo University China; ^3^ College of Food and Pharmaceutical Sciences Ningbo University China; ^4^ Zhejiang Collaborative Innovation Center for High Value Utilization of Byproducts from Ethylene Project Ningbo Polytechnic College China

**Keywords:** crystal structure, electrostatic potential, ferritin, metal ion movement, *Phascolosoma esculenta*

## Abstract

For marine invertebrates with no adaptive immune system, ferritin is a major intracellular iron‐storage protein with a critical role in innate immunity. Here, we present the crystal structures of two novel ferritins [Fer147 and *Phascolosoma esculenta* ferritin (PeFer)] from the marine invertebrate *P. esculenta*, which resides in muddy‐bottom coastal regions. Fer147 and PeFer exhibit the 4‐3‐2 symmetry of cage‐like hollow shells containing 24 subunits, similar to other known ferritins. Fer147 and PeFer contain both the conserved ferroxidase center and threefold channels. Subtle structural differences in the putative nucleation sites suggest possible routes of metal ion movement in the protein shells. However, the marked variation in the electrostatic potential of the threefold channels in Fer147 and the fourfold channels in PeFer suggests significant diversity between Fer147 and PeFer in terms of metal ion aggregation and cation exclusion. In summary, the presented crystal structures may serve as references for studies of the iron‐storage mechanism of additional ferritins from marine invertebrates.

## Graphical abstract

In this study, we present the crystal structures of two novel ferritins from the marine invertebrate *Phascolosoma esculenta*, which resides in muddy‐bottom coastal regions. They contain both the conserved ferroxidase center and 3‐fold channels, but exhibit marked variation in the electrostatic potential of the 3‐fold channels in Fer147 and the 4‐fold channels in PeFer.

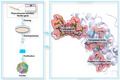

**Abbreviations**
ChF: *Chaetopterus* ferritin
H subunit: heavy subunit
HoLF: horse spleen L ferritin
HuHF: human ferritin H‐chain
HuLF: human ferritin L‐chain
L subunit: light subunit
M subunit: middle subunit
MjFer: *Marsupenaeus japonicus* ferritin
PeFer: *Phascolosoma esculenta* ferritin
pI: isoelectric point
ScFer: *Sinonovacula constricta* ferritin
SSRF: Shanghai Synchrotron Radiation Facility
TEM: transmission electron microscopy

Iron is an indispensable nutrient element for virtually all living organisms. Despite the crucial importance, excess iron poses a threat to all living organisms because of its pronounced toxicity in the presence of oxygen [1]. Ferritin is an intracellular iron‐storage protein that is ubiquitous in animal, plant, and microbial kingdoms. Ferritin is biologically available, sequesters iron in a nontoxic manner, and releases it when needed [2, 3]. Ferritin is also important for invertebrates. Invertebrates lack an adaptive immune system and rely solely on innate immunity to defend themselves from threatening environments where pathogenic microbes flourish [4, 5]. Similar to transferrin and lipocalin, ferritin effectively accumulates iron and plays a relevant role in innate immunity [6].

Structurally, members of the ferritin superfamily are usually spherical proteins composed of 24 polypeptide subunits that form a rhombic dodecahedron with twofold, threefold, and fourfold symmetry axes [7]. Ferritins are generally pierced by threefold and fourfold channels at their axis points, allowing iron or other small molecules to enter and exit the protein cage [8]. In particular, hydrophilic and hydrophobic pores across ferritin could be responsible for the entry and stable storage of iron [9]. The proteins have approximate interior and exterior diameters of 8 and 12 nm, respectively. They form a spherical polypeptide shell with a mass of 450–500 kDa surrounding a 6‐nm inorganic core of hydrated iron oxide ferrihydrite [3].

Ferritin is a conglomerate of identical or near‐identical subunits that act as building blocks for the assembly of a hollow interior cavity and spherical protein [10]. Three types of ferritin subunits have been identified in vertebrates: a light (L) subunit of ~ 19 kDa, a middle (M) subunit of ~ 20 kDa, and a heavy (H) subunit of ~ 21 kDa [11]. The H and L ferritins are found in higher vertebrates and M ferritins have been identified in lower vertebrates. The H and M subunits harbor a catalytic site for ferroxidase activity. Similar to the L subunit, the M subunit also contains an iron nucleation center [12, 13]. More than 50 different species of ferritin crystal structures have been deduced and deposited in the protein data bank. Very few are from marine invertebrates [12, 14]. Furthermore, the corresponding mechanisms underlying iron storage by ferritin in invertebrates are unclear.

Sipuncula *Phascolosoma esculenta* is a marine benthic invertebrate and member of the Sipuncula group. *P. esculenta* is unique to China and has a wide geographical distribution. *P. esculenta* is resistant to heavy metal pollution. The resistance has mainly been attributed to the ability of ferritins to chelate and sequester the toxic metals [15, 16]. Su *et al*. [17] first analyzed the structure of *P. esculenta* ferritin (PeFer) based on its cDNA sequence and successfully expressed recombinant PeFer. Ding *et al*. [16] discovered a novel protein interacting with PeFer using a yeast two‐hybrid system. The authors classified this protein, Fer147, as a number of several ferritin superfamilies. Herein, we determined the two crystal structures of *P. esculenta* and compared the crystal structures of PeFer and Fer147, and their roles in the assumed mechanism underlying iron storage by ferritins in *P. esculenta*.

## Materials and methods

### Cloning and expression

Based on the amino acid sequences of Fer147 [16] and PeFer [17], the theoretical isoelectric point (pI) and the predicted molecular weight were determined using the ExPASy server (http://web.expasy.org/peptide_mass/). Sequence alignment was performed using the ClustalW multiple sequence alignment program (https://www.genome.jp/tools‐bin/clustalw) and displayed using ESPript 3.0 [18].

Fer147 cDNA has been reported to be 916 bp in length with a 173 bp 5′‐UTR and 695 bp 3′‐UTR, with a 522 bp complete ORF encoding a polypeptide of 174 amino acids [16]. The cDNA of PeFer is 1017 bp in length with a 151 bp 5′‐UTR and 341 bp 3′‐UTR. It is comprised of a 525 bp complete ORF encoding a polypeptide of 174 amino acids. Gene‐specific primers encoding the mature polypeptide of ferritins were designed based on the sequence of Fer147 [16] and PeFer [17]. The primer sets for Fer147 (forward: 5′‐GCGCATATGATGTCTCTTTCGAGACCA‐3′; reverse: 5′‐GCGCTCGAGTCATTGAAGTTCTTCACT‐3′) and PeFer (forward: 5′‐GCCATATGGAAACGATGTCTCTGTCAAGA‐3′; reverse: 5′‐GCCTCGAGTTAGCTGTCGCCATC‐3′) contained *Nde*I and *Xho*I restriction sites at their 5′ ends, respectively. The amplified PCR fragments were cloned into a pMD18‐T vector (Takara, Dalian, China) and then digested using *Nde*I and *Xho*I (Takara) enzymes. They were subcloned into the *Nde*I/*Xho*I sites of the pET‐28a(+) expression vector (Novagen, Madison, WI, USA), and an N‐terminal His_6_‐SUMO tag was added to create recombinant Fer147 and PeFer plasmids [19]. The plasmids were transformed into *Escherichia coli* strain BL21(DE3) (Novagen).

### Protein purification

Cells were grown in Luria–Bertani medium (5 g yeast extract, 10 g tryptone, and 10 g NaCl per liter) containing 30 µg·mL^−1^ of kanamycin sulfate at 220 r.p.m. and 37 °C until the cell density reached an optical density at 600 nm (OD_600_) of 0.6. After induction for 18 h with 0.5 mm IPTG at 18 °C, the cells were harvested and resuspended in lysis buffer (25 mm Tris/HCl, pH 8.0, 150 mm NaCl, 0.5% (v/v) Triton X‐100). They were lysed by sonication and then centrifuged at 10 724 ***g*** for 30 min at 4 °C. The supernatant was recovered, and the proteins were then purified using the ӒKTA fast protein liquid chromatography system (GE Healthcare, Waukesha, WI, USA) with a Ni‐NTA affinity column (GE Healthcare) according to the manufacturer's instructions. After washing with dilution buffer A (25 mm Tris/HCl, pH 8.0, 150 mm NaCl, 70 mm imidazole), the bound proteins were eluted with dilution buffer B (25 mm Tris/HCl, pH 8.0, 150 mm NaCl, 500 mm imidazole). All purification steps were performed at 25 °C. The His_6_‐SUMO tags of the proteins were digested with SUMO protease overnight at 4 °C using a 1 : 500 ratio of protease to protein and removed using an Ni‐NTA affinity column (GE Healthcare). Subsequently, the purified proteins were concentrated to ~ 15 mg·mL^−1^ using a stirred ultrafiltration cell with a 30 kDa cutoff membrane filter (Millipore, Billerica, MA, USA) and stored at −80 °C. The purity of the ferritins was visualized using 12% SDS/PAGE. Concentrations of the purified proteins were determined using a bicinchoninic acid protein assay kit (Beyotime, Shanghai, China).

### Transmission electron microscopy (TEM)

The purified recombinant Fer147 and PeFer proteins were placed on carbon‐coated copper grids. After removing the excess solution with filter paper, the samples were negatively stained with 2% uranyl acetate for 5 min. TEM images were imaged at 80 kV using a model H‐7650 transmission electron microscope (Hitachi, Tokyo, Japan).

### Circular dichroism (CD) spectra

CD spectra were acquired using a model J‐715 CD spectropolarimeter (Jasco, Tokyo, Japan) with the far‐UV CD spectra range of 190–260 nm. The proteins were adjusted to a concentration of ~ 1.0 mg·mL^−1^ and loaded into a quartz cuvette with a 0.1 cm path length cell for measurement at room temperature. A binding buffer without protein was used for subtraction of the baseline signal. The nitrogen flow rate was 5 L·min^−1^, and the binding buffer was used as the reference solution. Typically, molar ellipticity [θ] (deg·cm^−2^·dmol^−1^) was calculated based on the mean amino acid residue weight of ferritins, as described by Ding *et al*. [16].

### Crystallization and structure determination

Preliminary screening for crystallization trials was conducted by the sitting‐drop vapor‐diffusion method using the Crystal Screen kit I and II (Hampton Research, Riverside, CA, USA) at 18 °C by mixing 1 µL of a 15 mg·mL^−1^ protein with a 1 : 1 ratio of a reservoir solution (Fer147: 0.1 m HEPES pH 7.5, and 20% (*v*/*v*) Jeffamine® M‐600®; PeFer: 0.1 m MES monohydrate pH 6.5, and 1.6 m MgSO_4_·7H_2_O). Each crystal was soaked in reservoir solutions containing 25% glycerol for 5 s, followed by flash‐freezing in liquid nitrogen.

All diffraction datasets of Fer147 and PeFer were collected at the BL17U1 [20] and BL19U1 [21] beamline of the Shanghai Synchrotron Radiation Facility (SSRF, Shanghai, China), respectively. Diffraction data were integrated, scaled, and merged using HKL‐2000 [22]. The initial phases of Fer147 and PeFer were determined through molecular replacement by the online version of BALBES, a molecular‐replacement pipeline [23]. Manual editing of the model was performed using the COOT [24]. Metal ions were positioned in the higher (*F*_o_−*F*_c_) residual densities and based on shorter bond distances with neighboring water molecules or other protein ligands [25]. The model refinement was carried out using the REFMAC5 program [26]. Occupancies of metal ions were manually adjusted to minimize the difference density. Superpositioning of Fer147 and PeFer with other structures was performed using the online server Superpose (http://superpose.wishartlab.com/) [27]. All figures were generated using the pymol molecular graphics system (Version 2.4.0, Schrödinger, LLC, New York, NY, USA). The mapping surface electrostatic potential was performed using the APBS application [28]. The data collection and structure refinement statistics are summarized in Table 1.

**Table 1:** Crystallographic statistics.

Crystal parameters	Fer147	PerFer
Data collection		
Space group	I222	I432
*a*, *b*, *c* (Å)	153.01, 153.49, 153.82	232.76, 232.76, 232.76
α, β, γ (˚)	90, 90, 90	90, 90, 90
Wavelength (Å)	0.960	0.979
Resolution range (Å)	34.25–1.65	41.15–1.99
Total no. of observations	217 040	159 439
No. of unique reflections	207 919	79 720
Completeness (%)	96.383	99.481
<*I*/σ(*I*)>	44.0 (2.9)^a^	21.6 (2.3)^a^
Wilson *B*‐factor (Å^2^)	13.3	33.3
*R*_merge_ (%)	1.3 (9.3)^a^	1.5 (29.5)^a^
CC_1/2_ (%)	100 (99.9)^a^	100 (69.0)^a^
Model refinement		
No. reflections used	197 507	68 702
*R*_work_	0.108	0.249
*R*_free_	0.153	0.302
RSMD		
R.m.s.d. bonds (Å)	0.029	0.013
R.m.s.d. angles (°)	2.60	1.54
Average *B*‐factor (Å^2^)	17.0	38.0
Ramachandran statistics		
Most favored regions (%)	98.95	98.20
Allowed regions (%)	1.05	1.80
Disallowed regions (%)	0.00	0.00
PDB code	6LPD	6LPE

^a^ Highest resolution shell is shown in parentheses.

## Results

**Fig. 1:**
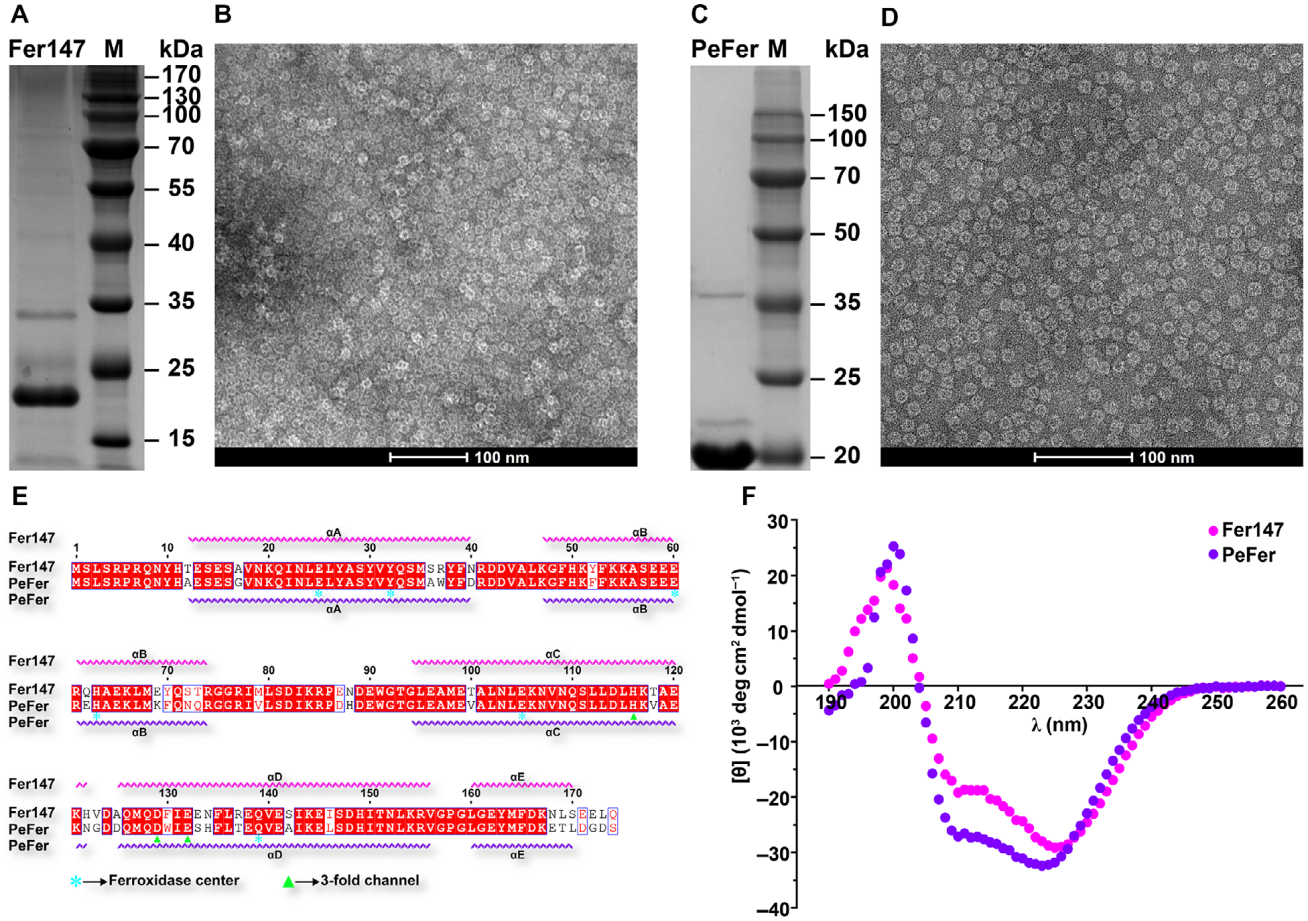
(A) Purification of Fer147 by SDS/PAGE analysis. (B) TEM images of recombinant Fer147. The scale bar denotes 100 nm. (C) Purification of PeFer by SDS/PAGE analysis. (D) TEM images of recombinant PeFer. The scale bar denotes 100 nm. (E) Sequence alignment of Fer147 and PeFer. (F) CD spectrum and secondary structure of Fer147 and PeFer.

The calculated molecular masses of Fer147 and PeFer without the signal peptide were 20.398 and 20.196 kDa, respectively, with a respective theoretical pI of 5.32 and 5.08. SDS/PAGE analysis revealed a molecular weight of ~ 20 kDa for both the recombinant Fer147 and PeFer subunits (Fig. 1A,C). The spherical features of Fer147 and PeFer were confirmed by TEM analysis (Fig. 1B,D). Multiple sequence alignment indicated that Fer147 was very similar to PeFer (GenBank accession number: ABW75858.1), with 80.8% sequence identity over 174 amino acids (Fig. 1E). Moreover, they each contained seven conserved amino acid residues in the ferroxidase center and three conserved amino acid residues of the threefold channel. CD analysis revealed that Fer147 and PeFer were α‐helical, resulting in a maximum at 195 nm and well‐defined minima at 208 and 225 nm (Fig. 1F).

**Fig. 2:**
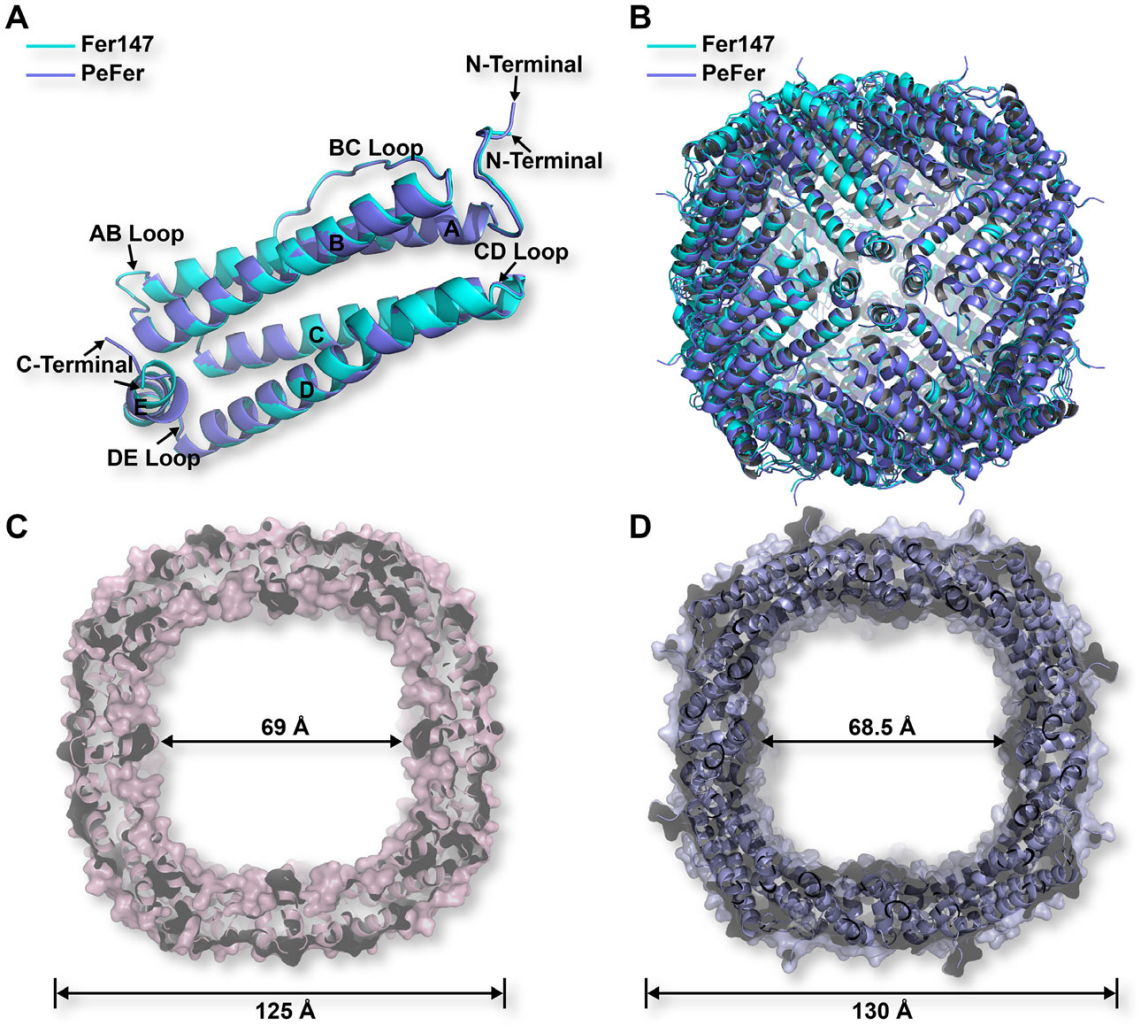
Structure superimposition for monomeric ferritin subunit of Fer147 and PeFer (A). Structure superimposition for ferritin cages of Fer147 and PeFer (B). The cross section image of the cage‐like cavity of the Fer147 (C) and PeFer (D).

**Fig. 3:**
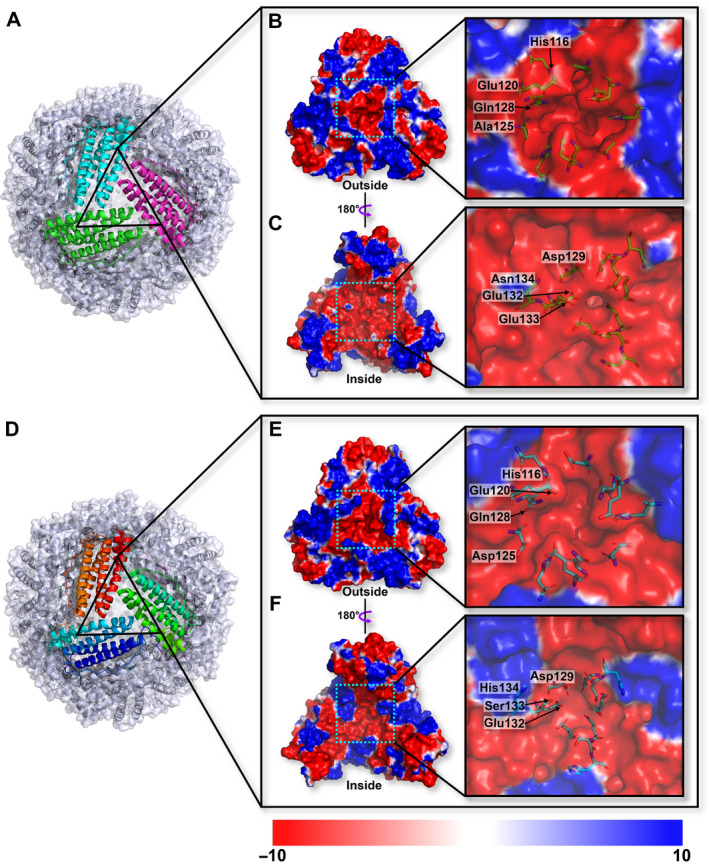
Overall structure of Fer147 (A) and PeFer (D) viewed from the 3‐fold channel. The surface electrostatic potential of Fer147 viewed from the outer (B, E) and inner (C, F) side of the 3‐fold channel. The potential scale is rendered from the −10 to +10 kT·e^−1^ from red to blue.

**Fig. 4:**
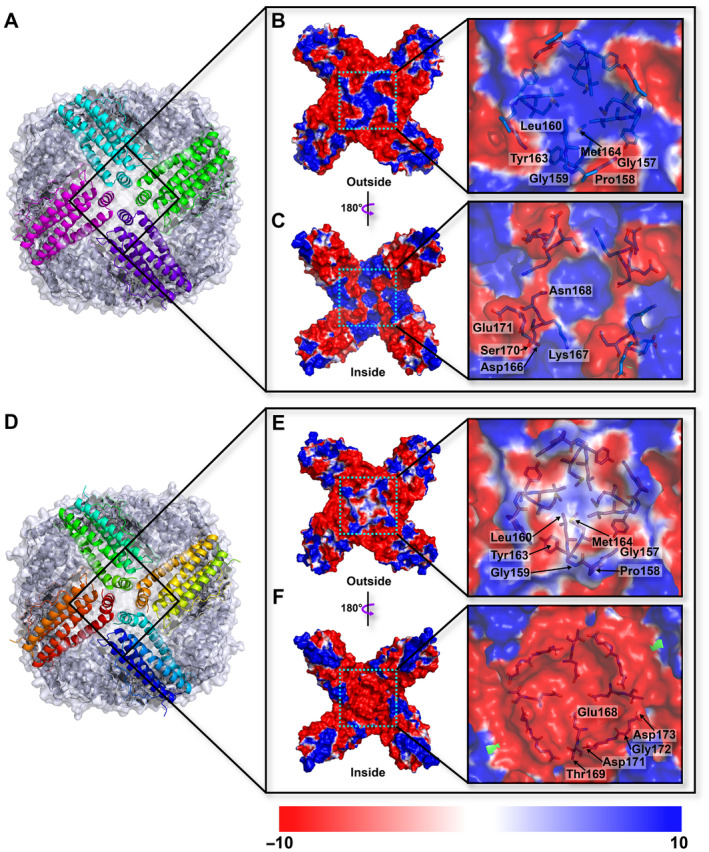
Overall structure of Fer147 (A) and PeFer (D) viewed from the 4‐fold channel. The surface electrostatic potential of PeFer viewed from the outer (B, E) and inner (C, F) side of the 4‐fold channel. The potential scale is rendered from the −10 to +10 kT·e^−1^ from red to blue.

The X‐ray diffraction data of Fer147 and PeFer were collected with an up to 1.65 and 1.99 Å resolution, respectively (Table 1). The 24 independent chains of Fer147 and PeFer each contain 170 and 172 residues, with five α‐helices (αA–αE) in each. Adjacent pairs of subunits are essentially orthogonal to one another in an assembled nanocage (Fig. 2A). Superimposition of the structures of Fer147 and PeFer revealed the good fit with the corresponding elements (Fig. 2B). The RMSD of Fer147 and PeFer monomers was 0.56 Å over 169 Cα atoms. A typical ferritin subunit structure of the Fer147 and PeFer monomers (Fig. 2A,B) comprised residues 12–40 (helix A), 47–74 (helix B), 94–122 (helix C), 125–156 (helix D), and 160–170 (helix E) (Fig. 1E). The protein shells of Fer147 and PeFer assembled into a large spherical cage with a respective inner diameter of 69 and 68.5 Å, and respective outer diameter of 125 and 130 Å (Fig. 2C,D). The symmetric arrangement of subunits from ferritins can form eight and six channels at the threefold and fourfold symmetry axes through the protein coat, respectively (Figs 3 and 4). The threefold channels form where three subunits come into contact through the N‐terminal end of helix D and the C‐terminal end of helix C (Fig. 3A,D). The fourfold channels form at the encounter point between the C‐terminals of helix E of four subunits from different pairs. These four symmetry‐related E‐helices determine the inner wall of the channel (Fig. 4A,D).

Based on the calculation of the electrostatic potentials of these surfaces, the threefold channels in both Fer147 and PeFer give rise to a funnel‐like pore that is mainly lined with negatively charged amino acids to form a negative electrostatic potential (Fig. 3). However, compared with PeFer, the threefold channel in Fer147 presents a pronounced preponderance of the negative electrostatic potential region from the overall view of the protein inner cavity plane (Fig. 3C,F). Comparison of these amino acid residues forming a threefold channel showed that most amino acids of the outer and inner entries are conserved in Fer147 and PeFer. However, in the threefold channel, the outer entry is included in Ala125 of Fer147 and Asp125 of PeFer (Fig. 3B,E). The inner entry is lined with Glu133 and Asn134 in Fer147, corresponding to Ser133 and His134 in PeFer (Fig. 3C,F). In contrast to the threefold channel, the channel along the fourfold symmetry axes of Fer147 and PeFer protein coats forms a hydrophobic and tightly packed channel lined with neutrally and positively charged residues (Fig. 4B,E). Nevertheless, the inner cavity from the fourfold channel in PeFer is dominated by the distribution of the negative electrostatic potential region, which is quite distinct from the fourfold channels in Fer147 (Fig. 4C,F). In contrast to Asn168, Glu171, and Leu173 in Fer147, the fourfold channel of PeFer near the inner cavity is lined with three acidic residues (Glu168, Asp171, and Asp173) (Fig. 1E).

**Fig. 5:**
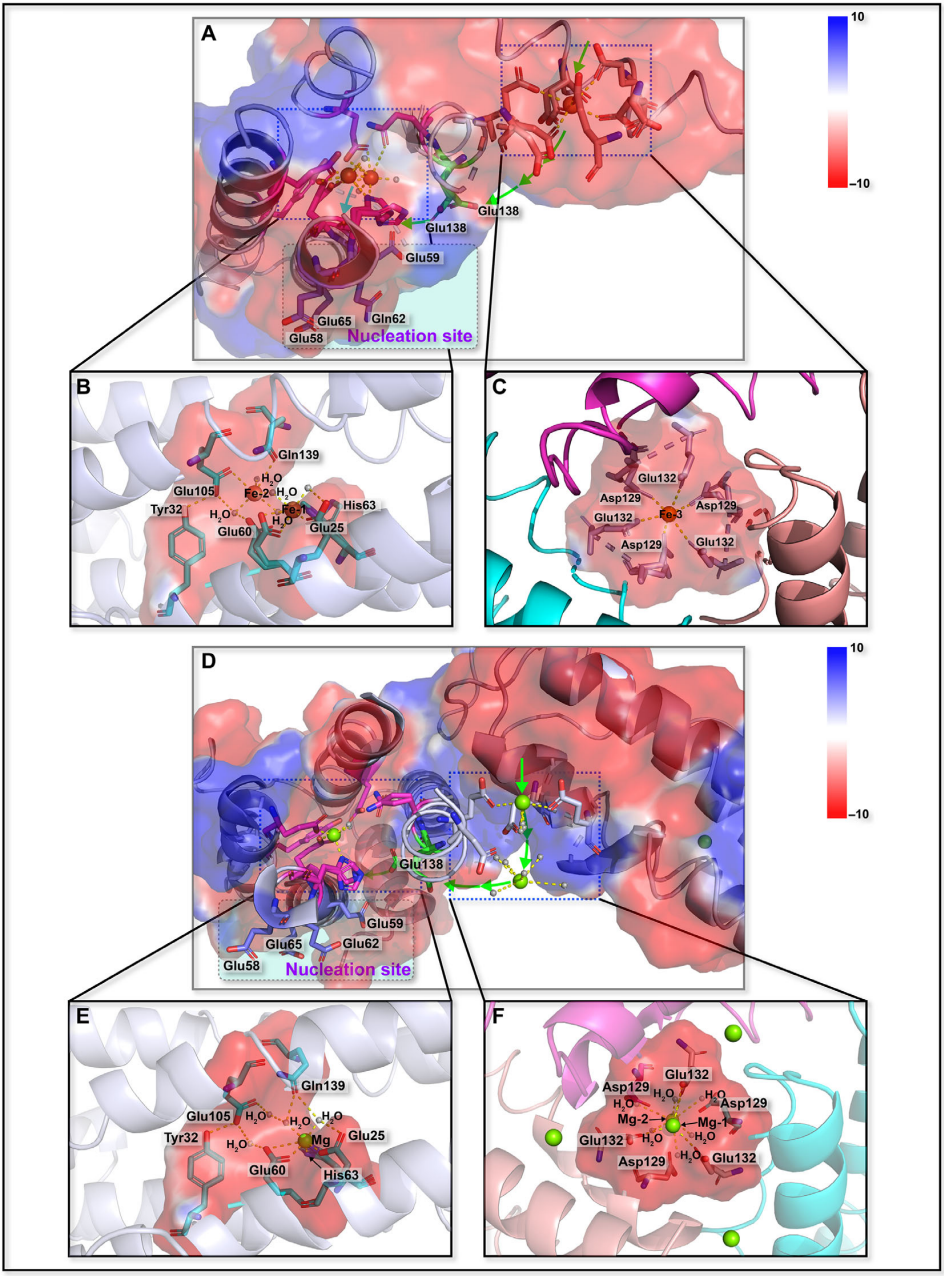
Schematic overview of the trajectory of metal ion moving through ferritin external pores to the threefold channel and to the ferroxidase sites, and subsequently into the nucleation site of Fer147 (A) and PeFer (D). The detailed structure of the ferroxidase site (B, E) and the ion entry channel (C, F) of Fer147 and PeFer.

For Fer147 and PeFer, six conserved amino acid residues (Glu25, Tyr32, Glu60, His63, Glu105, and Gln139) were identified in the ferroxidase center. The residues conferred the electron density of binuclear iron sites (Fig. 5B,E). In Fer147, each monomer binds with two iron cations, and the two metal binding sites were designated Fe‐1 and Fe‐2. The iron ion bound at the Fe‐1 site in Fer147 is ligated by two monodentate glutamate residues (Glu25 and Glu60), one histidine (His63) residue, and three water molecules. The iron ion at site Fe‐2 is ligated by two monodentate glutamate residues (Glu25 and Glu105), a glutamine (Gln139) residue, and four water molecules (Fig. 5B). However, only one magnesium ion bound at the Fe‐1 site in PeFer is ligated by two monodentate glutamate residues (Glu25 and Glu60), a bridging histidine (His63) residue and two water molecules (Fig. 5E). The Fe‐3 bound at the threefold channel in Fer147 is ligated by monodentate aspartate (Asp129) and glutamate (Glu132) residues and one water molecule (Fig. 5C). Moreover, two magnesium ions appear in PeFer, and only Mg‐1 ion binds to glutamate (Glu132) and three water molecules (Fig. 5F). Compared with the putative nucleation sites of Glu58, Glu59, Gln62, and Glu65 in Fer147, these sites facing the inner cavity of PeFer are included in four glutamate (Glu58, Glu59, Glu62, and Glu65) residues (Fig. 5A,D).

## Discussion

In this study, we heterologously expressed, purified, and crystallized the Fer147 and PeFer ferritins in *P. esculenta*. The crystal structures of Fer147 and PeFer display a cage‐like hollow spherical shell composed of 24 subunits. This assembly is similar to most ferritins from eukaryotes and prokaryotes [3]. However, the detailed structural comparison revealed that Fer147 and PeFer displayed some significant differences from other ferritins.

Ferritin can be divided into various groups, including those of H, L, and M chains or mitochondrial and serum ferritins [11]. Masuda *et al*. [13] described that the *Marsupenaeus japonicus* ferritin (MjFer) (PDB ID: 6A4U) subunit resembled the main characteristics of vertebrate H and L ferritins. Moreover, De Meulenaere *et al*. [12] indicated that the ferroxidase reaction rate of marine invertebrate *Chaetopterus* ferritin (ChF) (PDB ID: 5WPN) was eightfold faster than that of recombinant human H‐chain ferritin (HuHF). Despite their origins from vastly different organisms, the main chain Cα RMSDs are 0.52 Å between Fer147 and MjFer for 168 residues (60.6% identity over 170 amino acids), 0.44 Å between Fer147 and ChF for 168 residues (71.2% identity over 170 amino acids), 0.63 Å between PeFer and MjFer for 168 residues (61.8% identity over 173 amino acids), and 0.38 Å between PeFer and ChF for 169 residues (73.8% identity over 172 amino acids). Furthermore, the main chain Cα RMSD values between Fer147 of HuHF (PDB ID: 2FHA [29], 61.6% identity over 172 amino acids) and that of the human ferritin L‐chain (HuLF) (PDB ID: 5LG8 [30], 47.1% identity over 174 amino acids) are both 0.72 Å over 169 and 168 residues, respectively. Also, the Cα RMSD values between the PeFer of HuHF and that of HuLF are 0.56 Å over 169 residues and 0.69 Å over 170 residues, respectively, with respective sequence identity of 64.0% and 51.4% over 174 residues. Therefore, we can speculate that both Fer147 and PeFer might function similar to these marine invertebrates containing the hybrid type of H and L ferritin.

Generally, the threefold hydrophilic channel is widely acknowledged to penetrate into the ferritin shell cavity by metal ions and metal complexes, while the fourfold channel is not used for metal penetration [3]. In the structures of Fer147 and PeFer, most amino acids composed of the threefold channel are highly conserved. They include Asp129 and Glu132 presenting in their narrowest area (Fig. 3C,F), consistent with previous reports [31]. Moreover, the threefold channels of Fer147 and PeFer are interlaced positive and negative regions of the electrostatic potential (Fig. 3). This arrangement can give rise to electrostatic fields that direct metal ions toward the channel entrance and provide a route of access to the core of ferritin via electrostatic attraction [32]. Notably, a comparison of the electrostatic potential of the threefold channel for the two ferritins indicated that Fer147 presented many more negative potential regions than PeFer (Fig. 3C,F). This finding suggests that Fer147 could form a larger region of pronounced negative potential surrounded by a threefold channel, resulting in accelerated aggregation of metal ion [33]. The fourfold channel is markedly diverse among different organisms [3]. However, since the fourfold channel is usually be lined mostly by hydrophobic residues (e.g., leucine) in vertebrates, it is not used for metal penetration [34]. Electrostatic potential analysis indicated a significant difference in the distribution of electrostatic potential along the fourfold channel of Fer147 and PeFer. It is worth noting that almost all the surfaces of the exterior entrance of the fourfold channel have positive electrostatic potential (Fig. 4C,F), which could be unfavorable for the entry of some cations into the fourfold channel [34]. This electrostatic distribution is mainly attributed to the four symmetrical C‐terminal of the E‐helices formed by several amino acid residues (Fig. 2). Of note, the fourfold channel forming the inner cavity of PeFer presently displayed completely negative potential (Fig. 4F), as was also observed by Khare *et al*. [35]. This result suggests a possible functional role of the fourfold channel in providing pathways that facilitate cation exclusion from inside the protein shell and core formation [36].

In this study, Fer147 and PeFer formed ferroxidase centers composed chiefly of six conserved amino acid residues and conserved ion channels as found in *Sinonovacula constricta* ferritin [19]. In the structure of Fer147, the ferroxidase center is generally composed of two metal binding sites, A and B [2, 13] (Fig. 5A,B), and one iron ion is observed inside the threefold channel and coordinated with Asp129 and Glu132 (Fig. 5C), corresponding to Asp131 and Glu134 of HuHF [33], and to Asp134 and Glu133 of MjFer [13], respectively. However, in the crystal structure of PeFer, both ferroxidase center and threefold channel are associated with some huge positive peaks in the *F*_o_−*F*_c_ map. Obviously, the nature of metal ions can be determined based not only on the electron density, but also on the contextual evidence from other sources, for example, protein solution at any purification or crystallization step [37]. In this study, the crystallization mother liquid in PeFer contained a higher concentration (1.6 m) of MgSO_4_. Therefore, the assignment of ions appeared reasonable as MgSO_4_ from the crystallization solution in the crystal structure of PeFer. Positions of magnesium ions were assigned on the basis of the relatively strong electron density and the octahedral coordination geometry, that is, the relatively short distance between magnesium ion and ligand (amino acid or water molecules) [38, 39]. In the present structure of PeFer, only one magnesium ion is bound to Glu25, Glu60, His63, and two water molecules in the ferroxidase center (Fig. 5D,E), as previously observed for other ferritins [40, 41]. Two magnesium ions are present in the threefold channel where Mg‐1 ion is coordinated with the side chain of Glu132 and three water molecules, while Mg‐2 ion that stands exactly in the cavity of threefold channel is coordinated with six water molecules (Fig. 5D,F). Moreover, it has been reported that the conserved Glu139 with double conformation could flexibly translocate iron from the entry channel to the ferroxidase site with the assistance of the double conformation of His63 [13]. Thus, we infer that Glu138 with double conformation can play a crucial role in the transit site, and further research can be expended by site‐directed mutagenesis experiment to elucidate the role of this flexible side chain in the structures of Fer147 and PeFer. Accordingly, it can be concluded that in Fer147 and PeFer, metal ions are attracted by the specific array of carboxylate groups of Asp129 and Glu132 from the outside environment into the cage and are then transferred from the threefold channel to the ferroxidase site with the assistance of Glu138 (Fig. 5A,D). The putative nucleation sites of Fer147 are composed of Glu58, Glu59, Gln62, and Glu65, whereas in PeFer they are composed of four glutamate residues (Glu58, Glu59, Glu62, and Glu65), corresponding to Glu53, Glu56, Glu57, and Glu60 of the HuLF and horse spleen L ferritin (HoLF) [29, 30]. Therefore, although Glu57 of HuLF and HoLF is substituted by Gln62 in Fer147, the relative positions of these residues are well conserved and structurally analogous to those L ferritins.

## Conclusions

This study provides the first complete structural characterization of Fer147 and PeFer from marine invertebrate *P. esculenta* at 1.65 and 1.99 Å resolution, respectively. Both Fer147 and PeFer exhibit the 4‐3‐2 symmetry of cage‐like hollow shells containing 24 subunits commonly found in other known ferritins. A larger region of very negative potential is surrounded by a threefold channel in Fer147, suggesting an acceleration of metal ions aggregation. The fourfold channel of PeFer has a significantly different electrostatic potential arrangement from that of Fer147, suggesting that this channel may function as a possible pathway facilitating cation exclusion from inside of protein shells. Structural comparison revealed that Fer147 and PeFer contain both the conserved ferroxidase center and threefold channel, providing a structural insight into the possible routes of metal ion movement in the protein shells.

## Conflict of interest

The authors declare no conflict of interest.

## Author contributions

CL and XS conceived and designed the experiments. TM, HH, CS, CH, YW, QJ, and XQ collected and analyzed the data. TM performed structure determination, wrote an original draft. TM, CL, JZ, YL, and XS made manuscript revisions. All authors contributed to the final manuscript.

## Data Availability

The atomic coordinates and structural factors of Fer147 and PeFer have been deposited in the PDB under the accession code 6LPD and 6LPE, respectively.

## References

[1] Galaris D and Pantopoulos K (2008) Oxidative stress and iron homeostasis: mechanistic and health aspects. Crit Rev Clin Lab Sci 45, 1–23. 18293179 10.1080/10408360701713104

[2] Carrondo MA (2003) Ferritins, iron uptake and storage from the bacterioferritin viewpoint. EMBO J 22, 1959–1968. 12727864 10.1093/emboj/cdg215 PMC156087

[3] Aronovitz N , Neeman M and Zarivach R (2016) Ferritin iron mineralization and storage: from structure to functioned. In Iron Oxides: From Nature to Applications ( Faivre D , ed), pp. 117–142.Wiley‐VCH Verlag GmbH & Co. KGaA, Weinheim.

[4] Cassat JE and Skaar EP (2013) Iron in infection and immunity. Cell Host Microbe 13, 509–519. 23684303 10.1016/j.chom.2013.04.010 PMC3676888

[5] Ong ST , Ho JZ , Ho B and Ding JL (2006) Iron‐withholding strategy in innate immunity. Immunobiology 211, 295–314. 16697921 10.1016/j.imbio.2006.02.004

[6] Kong P , Wang L , Zhang H , Zhou Z , Qiu L , Gai Y and Song L (2010) Two novel secreted ferritins involved in immune defense of Chinese mitten crab *Eriocheir sinensis* . Fish Shellfish Immunol 28, 604–612. 20045469 10.1016/j.fsi.2009.12.018

[7] Theil EC , Matzapetakis M and Liu X (2006) Ferritins: iron/oxygen biominerals in protein nanocages. J Biol Inorg Chem 11, 803. 16868744 10.1007/s00775-006-0125-6

[8] Watt RK , Hilton RJ and Graff DM (2010) Oxido‐reduction is not the only mechanism allowing ions to traverse the ferritin protein shell. Biochim Biophys Acta 1800, 745–759. 20214952 10.1016/j.bbagen.2010.03.001

[9] Thiruselvam V , Sivaraman P , Kumarevel T and Ponnuswamy MN (2014) Revelation of endogenously bound Fe^2+^ ions in the crystal structure of ferritin from *Escherichia coli* . Biochem Biophys Res Commun 453, 636–641. 25305494 10.1016/j.bbrc.2014.10.007

[10] Crichton RR and Declercq JP (2010) X‐ray structures of ferritins and related proteins. Biochim Biophys Acta 1800, 706–718. 20363295 10.1016/j.bbagen.2010.03.019

[11] Giorgi A , Mignogna G , Bellapadrona G , Gattoni M , Chiaraluce R , Consalvi V , Chiancone E and Stefanini S (2008) The unusual co‐assembly of H‐ and M‐chains in the ferritin molecule from the Antarctic teleosts *Trematomus bernacchii* and *Trematomus newnesi* . Arch Biochem Biophys 478, 69–74. 18625196 10.1016/j.abb.2008.06.022

[12] De Meulenaere E , Bailey JB , Tezcan FA and Deheyn DD (2017) First biochemical and crystallographic characterization of a fast‐performing ferritin from a marine invertebrate. Biochem J 474, 4193–4206. 29127253 10.1042/BCJ20170681

[13] Masuda T , Zang J , Zhao G and Mikami B (2018) The first crystal structure of crustacean ferritin that is a hybrid type of H and L ferritin. Protein Sci 27, 1955–1960. 30099791 10.1002/pro.3495 PMC6201719

[14] Rose PW , Prlić A , Altunkaya A , Bi C , Bradley AR , Christie CH , Costanzo LD , Duarte JM , Dutta S , Feng Z *et al*. (2017) The RCSB protein data bank: integrative view of protein, gene and 3D structural information. Nucleic Acids Res 45, D271–D281. 27794042 10.1093/nar/gkw1000 PMC5210513

[15] Ding H , Zhou J , Han Y and Su X (2018) Characterization of recombinant *Phascolosoma esculenta* ferritin as an efficient heavy metal scavenger. Protein Pept Lett 404, 767–775. 10.2174/0929866525666180806111756 30081783

[16] Ding H , Zhang D , Chu S , Zhou J and Su X (2017) Screening and structural and functional investigation of a novel ferritin from *Phascolosoma esculenta* . Protein Sci 26, 2039–2050. 28726294 10.1002/pro.3241 PMC5606535

[17] Su X , Du L , Li Y , Li T , Li D , Wang M and He J (2009) Production of recombinant protein and polyclonal mouse antiserum for ferritin from Sipuncula *Phascolosoma esculenta* . Fish Shellfish Immunol 27, 466–468. 19563895 10.1016/j.fsi.2009.06.014

[18] Robert X and Gouet P (2014) Deciphering key features in protein structures with the new ENDscript server. Nucleic Acids Res 42, W320–W324. 24753421 10.1093/nar/gku316 PMC4086106

[19] Su C , Ming T , Wu Y , Jiang Q , Huan H , Lu C , Zhou J , Li Y , Song H and Su X (2020) Crystallographic characterization of ferritin from *Sinonovacula constricta* . Biochem Biophys Res Commun 524, 217–223. 31983429 10.1016/j.bbrc.2020.01.069

[20] Wang Q‐S , Zhang K‐H , Cui Y , Wang Z‐J , Pan Q‐Y , Liu K , Sun B , Zhou H , Li M‐J , Xu Q , Xu C‐Y , Yu F and He J‐H (2018) Upgrade of macromolecular crystallography beamline BL17U1 at SSRF. Nucl Sci Tech 29, 68.

[21] Zhang W‐Z , Tang J‐C , Wang S‐S , Wang Z‐J , Qin W‐M and He J‐H (2019) The protein complex crystallography beamline (BL19U1) at the Shanghai Synchrotron Radiation Facility. Nucl Sci Tech 30, 170.

[22] Otwinowski Z and Minor W (1997) Processing of X‐ray diffraction data collected in oscillation mode. Methods Enzymol 276, 307–326. 10.1016/S0076-6879(97)76066-X 27754618

[23] Long F , Vagin AA , Young P and Murshudov GN (2008) BALBES: a molecular‐replacement pipeline. Acta Crystallogr 64, 125–132. 10.1107/S0907444907050172 PMC2394813 18094476

[24] Emsley P , Lohkamp B , Scott WG and Cowtan K (2010) Features and development of Coot. Acta Crystallogr D Biol Crystallogr 66, 486–501. 20383002 10.1107/S0907444910007493 PMC2852313

[25] Masuda T , Goto F , Yoshihara T and Mikami B (2009) Crystal structure of plant ferritin reveals a novel metal binding site that functions as a transit site for metal transfer in ferritin. J Biol Chem 285, 4049–4059. 20007325 10.1074/jbc.M109.059790 PMC2823546

[26] Murshudov GN , Skubák P , Lebedev AA , Pannu NS , Steiner RA , Nicholls RA , Winn MD , Long F and Vagin AA (2011) REFMAC5 for the refinement of macromolecular crystal structures. Acta Crystallogr D Biol Crystallogr 67, 355–367. 21460454 10.1107/S0907444911001314 PMC3069751

[27] Maiti R , van Domselaar GH , Zhang H and Wishart DS (2004) Superpose: a simple server for sophisticated structural superposition. Nucleic Acids Res 23, W590–W594. 10.1093/nar/gkh477 PMC441615 15215457

[28] Baker NA , Sept D , Joseph S , Holst MJ and McCammon JA (2001) Electrostatics of nanosystems: application to microtubules and the ribosome. Proc Natl Acad Sci USA 98, 10037–10041. 11517324 10.1073/pnas.181342398 PMC56910

[29] Hempstead PD , Yewdall SJ , Fernie AR , Lawson DM , Artymiuk PJ , Rice DW , Ford GC and Harrison PM (1997) Comparison of the three‐dimensional structures of recombinant human H and horse L ferritins at high resolution. J Mol Biol 268, 424–448. 9159481 10.1006/jmbi.1997.0970

[30] Pozzi C , Ciambellotti S , Bernacchioni C , Di Pisa F , Mangani S and Turano P (2017) Chemistry at the protein‐mineral interface in L‐ferritin assists the assembly of a functional (μ(3)‐oxo)Tris[(μ(2)‐peroxo)] triiron(III) cluster. Proc Natl Acad Sci USA 114, 2580–2585. 28202724 10.1073/pnas.1614302114 PMC5347543

[31] Arenas‐Salinas M , Townsend PD , Brito C , Marquez V , Marabolli V , Gonzalez‐Nilo F , Matias C , Watt RK , López‐Castro JD , Domínguez‐Vera J *et al*. (2014) The crystal structure of ferritin from *Chlorobium tepidum* reveals a new conformation of the 4‐fold channel for this protein family. Biochimie 106, 39–47. 25079050 10.1016/j.biochi.2014.07.019

[32] Chandramouli B , Bernacchioni C , Di Maio D , Turano P and Brancato G (2016) Electrostatic and structural bases of Fe^2+^ translocation through ferritin channels. J Biol Chem 291, 25617–25628. 27756844 10.1074/jbc.M116.748046 PMC5207259

[33] Douglas T and Ripoll DR (1998) Calculated electrostatic gradients in recombinant human H‐chain ferritin. Protein Sci 7, 1083–1091. 9605313 10.1002/pro.5560070502 PMC2144004

[34] Abe S , Ito N , Maity B , Lu CL , Lu DN and Ueno T (2019) Coordination design of cadmium ions at the 4‐fold axis channel of the apo‐ferritin cage. Dalton Trans 48, 9759–9764. 30993287 10.1039/c9dt00609e

[35] Khare G , Gupta V , Nangpal P , Gupta RK , Sauter NK and Tyagi AK (2011) Ferritin structure from *Mycobacterium tuberculosis*: comparative study with homologues identifies extended C‐terminus involved in ferroxidase activity. PLoS One 6, e18570. 21494619 10.1371/journal.pone.0018570 PMC3072985

[36] Takahashi T and Kuyucak S (2003) Functional properties of threefold and fourfold channels in ferritin deduced from electrostatic calculations. Biophys J 84, 2256–2263. 12668434 10.1016/S0006-3495(03)75031-0 PMC1302792

[37] Roy S , Saraswathi R , Chatterji D and Vijayan M (2008) Structural studies on the second *Mycobacterium smegmatis* Dps: invariant and variable features of structure, assembly and function. J Mol Biol 375, 948–959. 18061613 10.1016/j.jmb.2007.10.023

[38] Tosha T , Behera RK , Ng HL , Bhattasali O , Alber T and Theil EC (2012) Ferritin protein nanocage ion channels: gating by N‐terminal extensions. J Biol Chem 287, 13016–13025. 22362775 10.1074/jbc.M111.332734 PMC3339931

[39] Tosha T , Ng HL , Bhattasali O , Alber T and Theil EC (2010) Moving metal ions through ferritin‐protein nanocages from three‐fold pores to catalytic sites. J Am Chem Soc 132, 14562–14569. 20866049 10.1021/ja105583d PMC3211085

[40] Langlois d'Estaintot B , Santambrogio P , Granier T , Gallois B , Chevalier JM , Précigoux G , Levi S and Arosio P (2004) Crystal structure and biochemical properties of the human mitochondrial ferritin and its mutant Ser144Ala. J Mol Biol 340, 277–293. 15201052 10.1016/j.jmb.2004.04.036

[41] Pozzi C , Di Pisa F , Lalli D , Rosa C , Theil E , Turano P and Mangani S (2015) Time‐lapse anomalous X‐ray diffraction shows how Fe^2+^ substrate ions move through ferritin protein nanocages to oxidoreductase sites. Acta Crystallogr D Biol Crystallogr 71, 941–953. 25849404 10.1107/S1399004715002333 PMC4388269

